# Supplementary material for: Machine learning of genomic features in organotropic metastases stratifies progression risk of primary tumors
Source: Nat Commun. 2021 Nov 18;12:6692. doi: 10.1038/s41467-021-27017-w (PMC8602327; doi:10.1038/s41467-021-27017-w)
Supplement: Supplementary file 1 — Supplementary Information [file 41467_2021_27017_MOESM1_ESM.pdf]

Figure S1: Clinical and genomic data collection and integration.

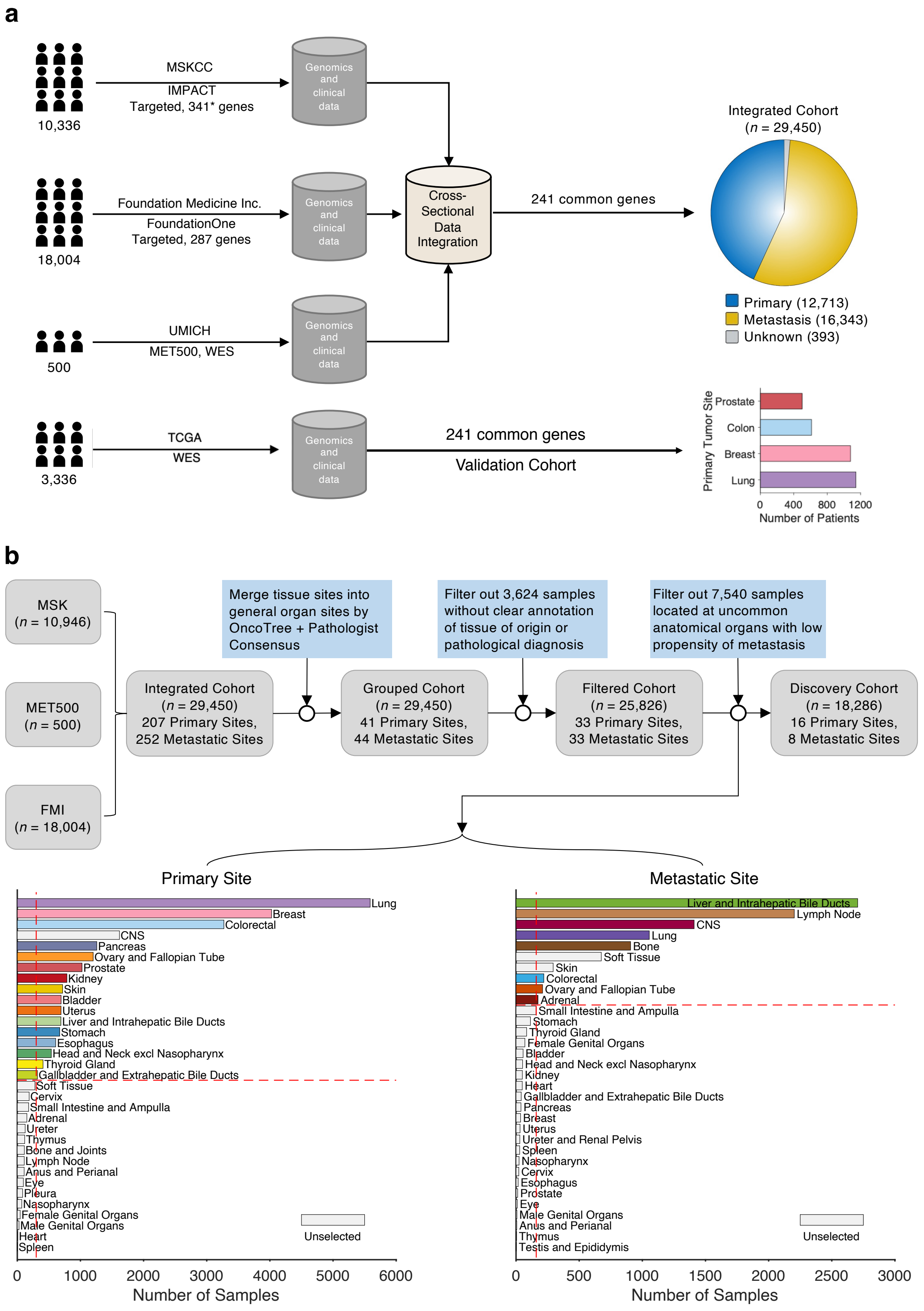

Supplementary Fig. 1 Clinical and genomic data collection and integration. a. Data collection and integration workflow. * Some MSK samples were sequenced by the panel of 468 genes. **b.** Workflow of cancer classification, sample filtering and selection. Red dashed lines denote the cutoffs used in pre-selection. Bars in light grey denote unselected cancer organs.

Figure S2: Comparison analysis of primary and metastasis samples.

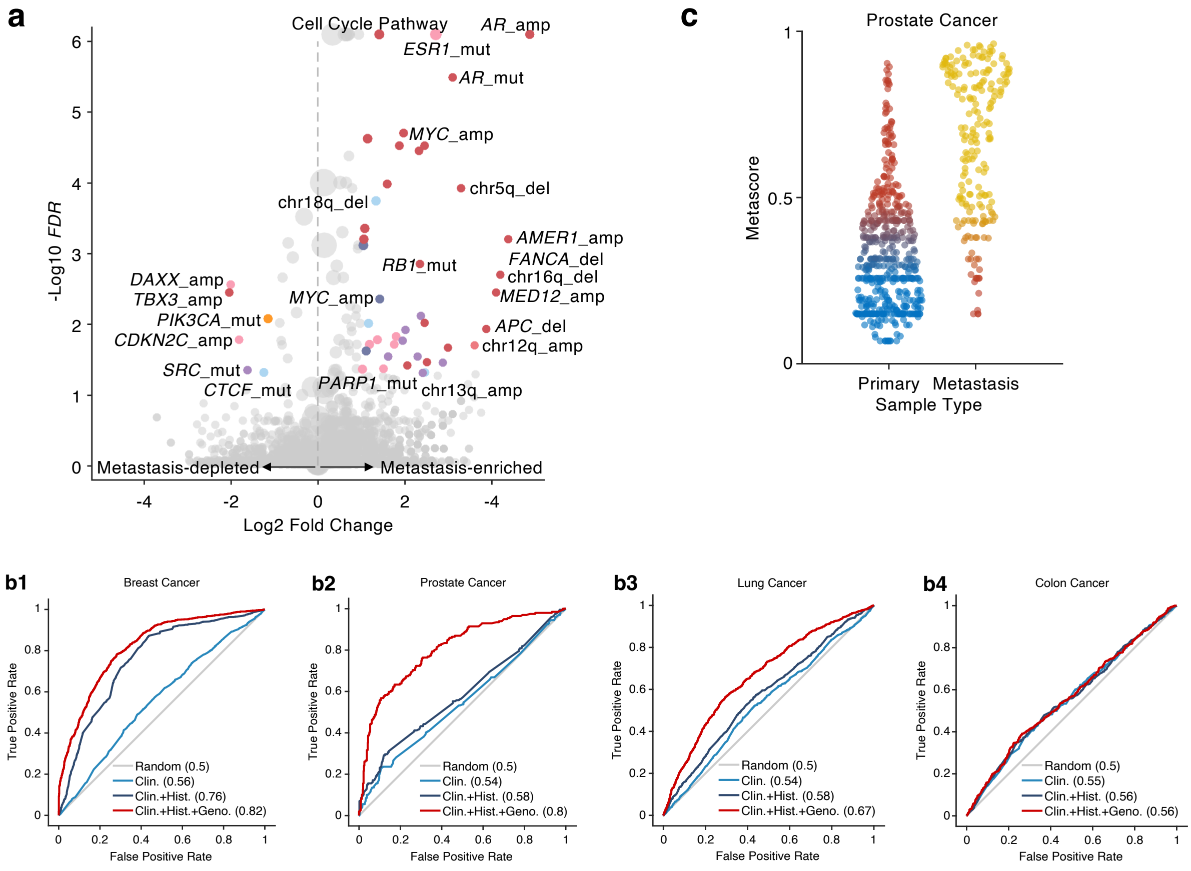

Supplementary Fig. 2 Comparison analysis of primary and metastasis samples. **a**. Differential analysis of variant fractions in metastatic versus primary cancer samples in the 16 cancer types. Node color represents the cancer type with identical color code used in **Fig. 1a**. Node size is proportional to the variant fraction in all the samples of one cancer type. Fold change is calculated by the variant fraction in metastasis over that in primary. Significance was estimated by proportion test with Benjamini-Hochberg correction. **b1-4**. Receiver operating characteristic (ROC) curves in separating clinically diagnosed metastasis from primary cancers by three models using clinical (Clin.), clinical plus histological (Clin. + Hist.), and clinical, histological plus genomic (Clin. + Hist. + Geno.) features in breast (**b1**), prostate (**b2**), lung (**b3**), and colon (**b4**) cancers. **c**. Metascore distribution in primary and metastatic prostate cancer, respectively.

Figure S3: Expression and clinical signatures of Metastasis-Featuring Primary (MFP).

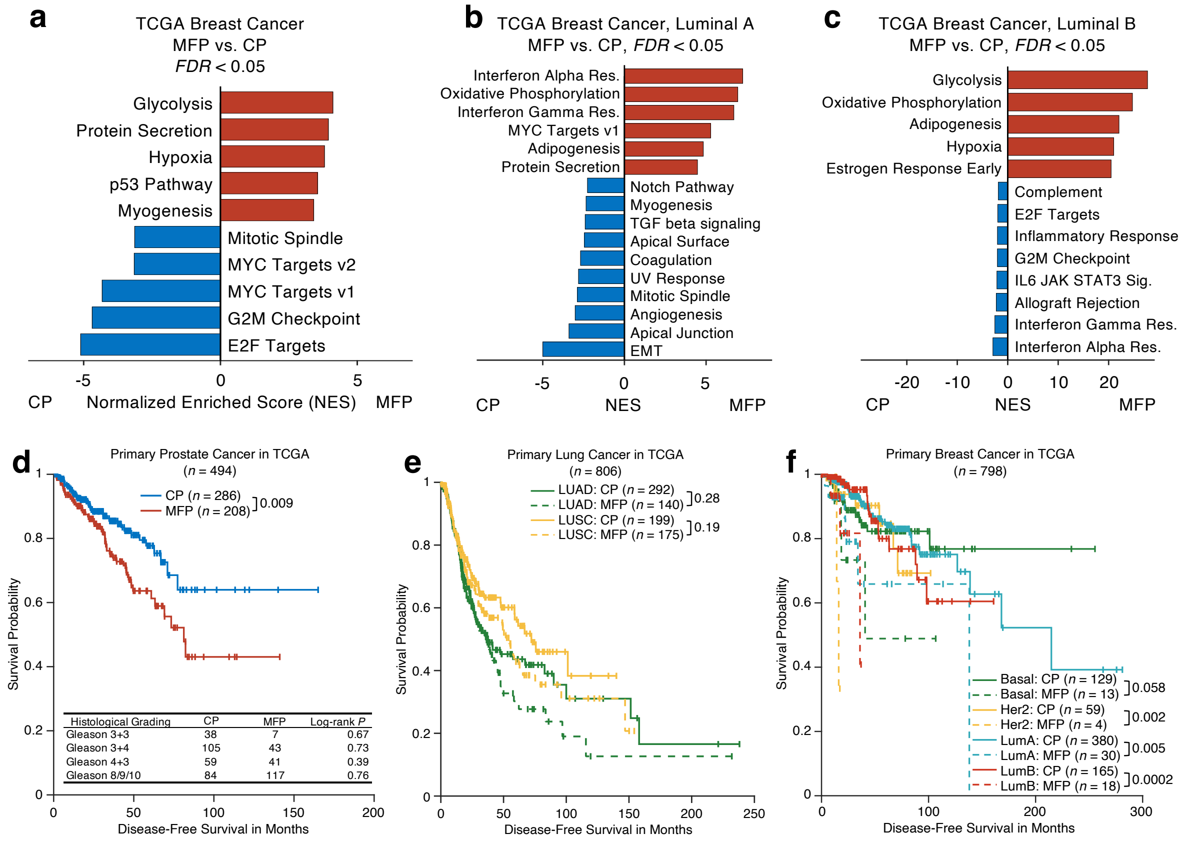

Supplementary Fig. 3 Expression and clinical signatures of Metastasis-Featuring Primary (MFP). a-c. Gene Set Enrichment Analysis (GSEA) in comparison of MFP tumors versus Conventional Primary (CP) tumors within all TCGA breast cancer samples (**a**), luminal A subtype (**b**), and luminal B subtype (**c**), *FDR* < 0.05. Res. is the abbreviation of Response. Sig. is the abbreviation of Signaling. **d-f**. Kaplan-Meier plot in comparison of disease-free survival (DFS) between MFP tumors versus CP tumors within TCGA prostate cancer cohort (**d,** the table lists the comparisons of DFS within four different histological grades of Gleason system), TCGA lung cancer cohort (**e**, LUAD: lung adenocarcinoma in green and LUSC: lung squamous cell carcinoma in yellow), and TCGA breast cancer cohort in four different molecular subtypes (**f**, Basal-like (Basal) in green, HER2-enriched (Her2) in yellow, Luminal A (LumA) in blue, and Luminal B (LumB) in red). Solid line denotes low-risk group with CP tumors, while dashed line denotes high-risk group with MFP tumors. All integers (*n*) denote sample size of the group. All decimals between two groups denote *p* values estimated in two-sided log-rank test.

Figure S4: Frequency of Metastatic sites in different datasets.

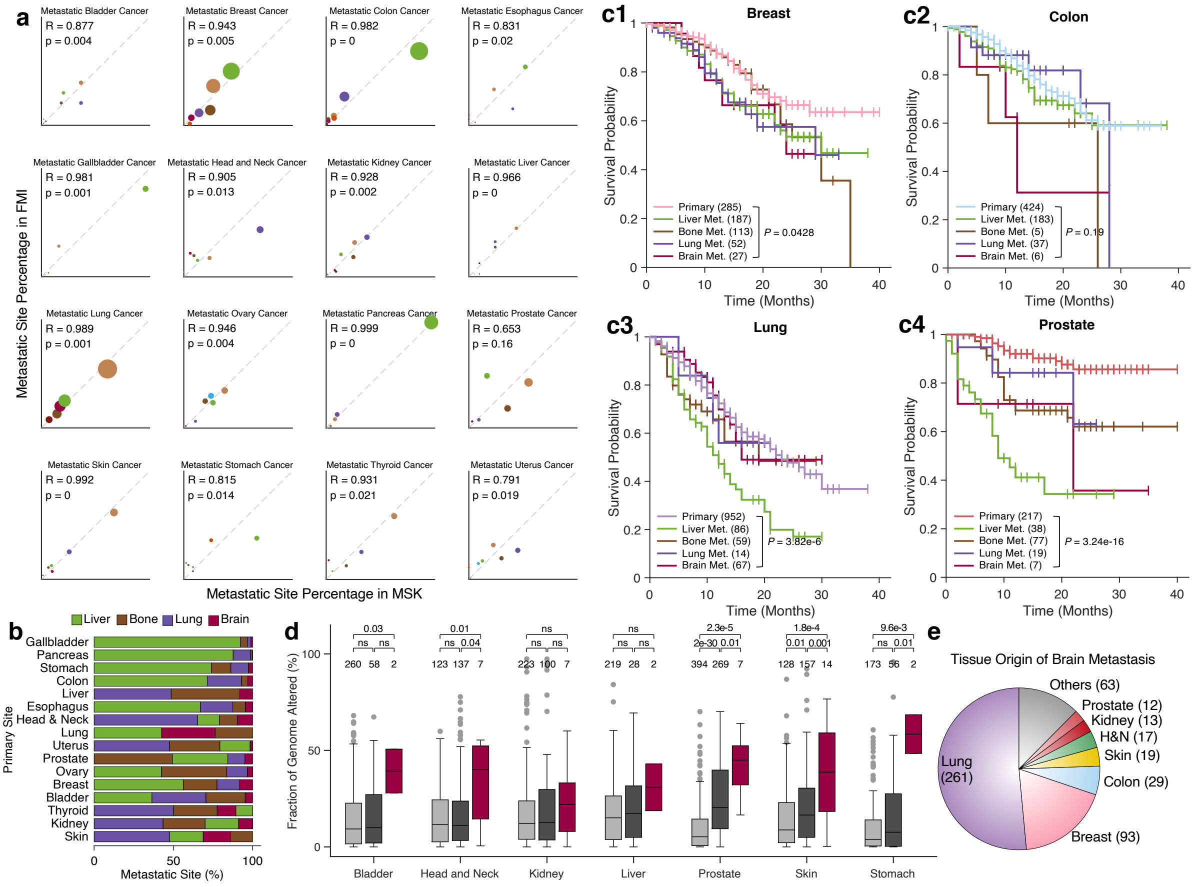

Supplementary Fig. 4 Frequency of Metastatic sites in different datasets. a. Correlations of metastatic site fractions between metastatic cancers in MSK cohort and FMI cohort in the 16 cancer types, respectively. R (Rho) value and its significance, *p* value, are derived from Pearson correlation analysis. **b**. Fractions of metastases at liver, bone, lung, and brain in the 16 cancers. **c**. Kaplan-Meier plots in comparison of primary and four metastatic cancers at bone, brain, liver, and lung within breast cancer (**c1**), colon cancer (**c2**), lung cancer (**c3**) and prostate cancer (**c4**), respectively. The significance is derived from two-sided log-rank test. **d**. Comparison of Fractions of Genome Altered (FGA) between primary, non-brain metastasis, and brain metastasis samples within the other seven cancer types not shown in **Fig. 4d**. respectively. Boxes represent upper and lower quartiles; the lines inside denote median; whiskers correspond to 1.5 times the interquartile range. Number of samples are denoted on top of boxes. Significance is derived from one-sided rank-sum test. ns: *p* >= 0.05. **e**. Tissue origin fraction of brain metastasis in the combined cohort of MSK, FMI and MET500. Number in the bracket denotes the number of samples.

Figure S5: Molecular signatures of breast and lung cancers brain metastases.

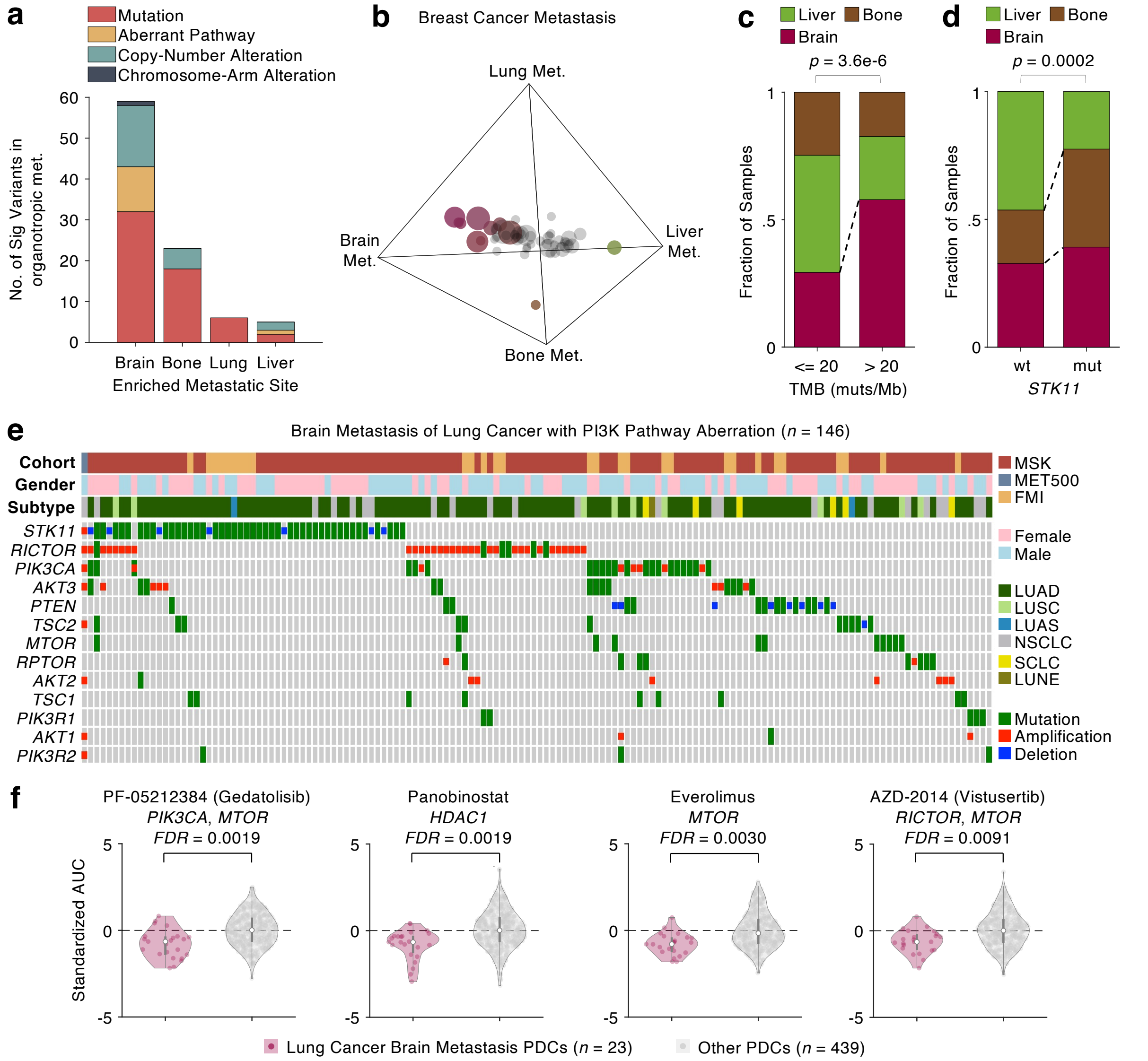

Supplementary Fig. 5 Molecular signatures of breast and lung cancers brain metastases. a. Number of variation types of 93 significant variants enriched in organotropic metastases at brain, bone, lung and liver. See Supplementary Data S3. **b**. Odds ratios of variant fraction at one site over that not at the site (OGTOR), for bone, brain, liver and lung metastases of breast cancer, projected in a tetragon space. Nodes highlighted in color are the variants with *FDR* < 0.05 (two-sided Chi-squared test) and variant fraction in the four sites > 5%. **c**-**d**. Variant fraction comparison within the liver, bone and brain metastases of lung cancer for Tumor Mutation Burden (TMB) (**c**, high: > 20 mutations per Mb, versus low: < 20 mutations per Mb) and *STK11* (**d**, wt: wildtype versus mut: mutant). Significance was estimated by Chi-squared test. **e**. Clinical and mutational landscape of 146 brain metastases of lung cancer with PI3K pathway aberration. **f**. Comparison of drug efficacies in 23 lung cancer brain metastasis patient-derived cell lines (PDCs) versus 439 other PDCs from various cancer types using standardized area under the dose-response curves (AUC). Four drugs are shown with drug names, drug target genes, and significance derived by two-sample *t*-test, *FDR* < 0.05, Benjamini-Hochberg correction. Boxes represent upper and lower quartiles; the lines inside denote median; whiskers correspond to 1.5 times the interquartile range.

Figure S6: Molecular signatures of colon cancer brain metastases.

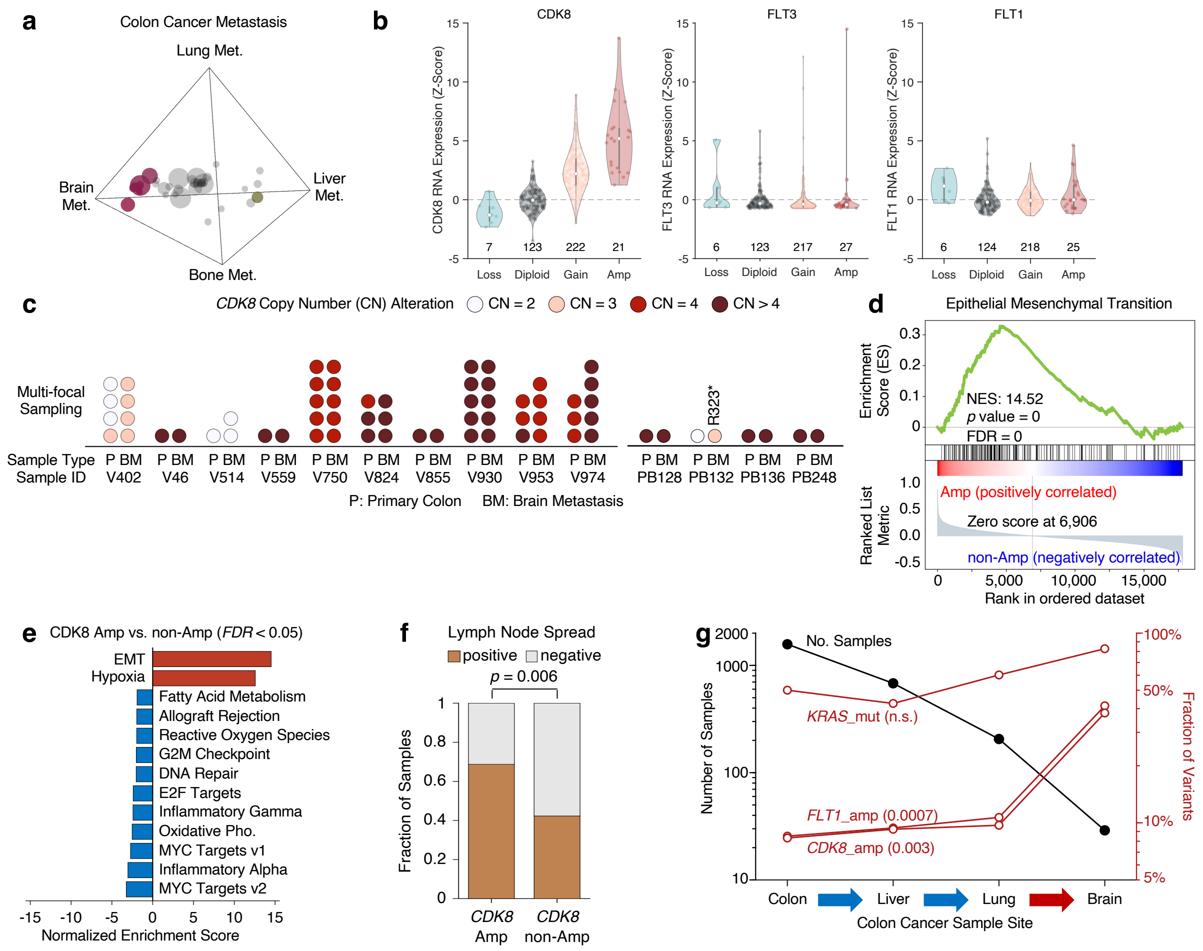

Supplementary Fig. 6 Molecular signatures of colon cancer brain metastases. a. Odds ratios of variant fraction at one site over that not at the site (OGTOR), for bone, brain, liver and lung metastases of colon cancer, projected in a tetragon space. Nodes highlighted in color are the variants with *FDR* < 0.05 (Chi-squared test) and variant fraction in the four sites > 5%. **b**. Standardized expression at four different ploidies (loss, diploid, gain and amplification) of *CDK8*, *FLT3* and *FLT1* in TCGA colon cancer cohort. Boxes represent upper and lower quartiles; the lines inside denote median; whiskers correspond to 1.5 times the interquartile range. **c**. *CDK8* copy numbers in paired-primary-brain-metastasis samples of 14 colon cancer patients. Nodes at the same column denote multi-focal sampling at one tumor lesion. **d-e**. GSEA between *CDK8*-amplified versus non-amplified samples in TCGA colon cancer cohort highlights Epithelial Mesenchymal Transition as the top significant activated function (**d**), and cell-cycle proliferation as the significant deactivated function (**e**) in *CDK8*-amplified samples. Significance and multiple comparison are derived by GSEA using 1000-time permutations within the gene sets. **f**. Comparison of lymph node spread sample fractions between *CDK8*-amplified versus non-amplified samples in TCGA colon cancer cohort. Significance is derived by two-sided proportion test. **g**. Number of samples (black line) and fraction of variants (red lines: *KRAS* mutation, *FLT1* and *CDK8* amplifications) in colon cancer metastatic cascade from colon to liver, lung and then brain, eventually (blue arrow denotes vein and red denotes artery). Significance was derived from proportion trend test with predefined trend: colon > liver > lung > brain.

Supplementary Table Clinical and histological features extracted from the clinical and diagnostic records.

Cancer Type	Clinical and Histological Feature
Breast Cancer	Gender (Female/Male)
	Age
	Race (African/Asian/Caucasian/Hispanic)
	Histology (Ductal/Lobular)
Prostate Cancer	Age
	Race (African/Asian/Caucasian/Hispanic)
	Histology (Adenocarcinoma/Neuroendocrine)
Lung Cancer	Gender (Female/Male)
	Smoking Status (Former or Current/Never)
	Age
	Race (African/Asian/Caucasian/Hispanic)
	Histology (Adenocarcinoma/Squamous Cell Carcinoma)
Colon Cancer	Gender (Female/Male)
	Age
	Race (African/Asian/Caucasian/Hispanic)
	Location (Colon/Rectal)
	Histology (Adenocarcinoma/Mucinous)
