# Supplementary material for: Machine learning of genomic features in organotropic metastases stratifies progression risk of primary tumors
Source: Nat Commun. 2021 Nov 18;12:6692. doi: 10.1038/s41467-021-27017-w (PMC8602327; doi:10.1038/s41467-021-27017-w)
Supplement: Supplementary file 3 — Description of Additional Supplementary Files [file 41467_2021_27017_MOESM3_ESM.pdf]

Description of Additional Supplementary Files

File Name: Supplementary Data 1
Description: Mapping of raw tissue sites to general anatomic organs for primary tumor site.

File Name: Supplementary Data 2
Description: Mapping of raw tissue sites to general anatomic organs for metastatic site.

File Name: Supplementary Data 3
Description: 93 genomic variants enriched in organotropic metastases (FDR < 0.1 in two-sided Chi-squared test and variant fraction in bone, brain, liver and lung metastases larger than 1%).

File Name: Supplementary Data 4
Description: Gene intersect of MSK-IMPACT and FoundationONE panels.
